# Genome-Wide Linkage Mapping of Root System Architecture-Related Traits Under Drought Stress in Common Wheat (*Triticum aestivum* L.)

**DOI:** 10.3390/plants14193023

**Published:** 2025-09-30

**Authors:** Yirong Jin, Guiju Chen, Xiaodong Qiu, Fuyan Wang, Hui Jin, Liang Zhang, Cheng Liu, Jianjun Liu, Wenjing Li, Peng Liu

**Affiliations:** 1 Dezhou Academy of Agricultural Sciences, Dezhou 253015, China; jyr2014@163.com (Y.J.);; 2 Wheat Research Institute, Jining Academy of Agricultural Sciences, Jining 272000, China; 3 Department of Science and Technology of Shandong Province, Jinan 250101, China; 4 Institute of Forage and Grassland Sciences, Heilongjiang Academy of Agricultural Sciences, Harbin 150086, China; 5 Crop Research Institute, Shandong Academy of Agricultural Sciences, Jinan 250100, China; 6 College of Life Science, Langfang Normal University, Langfang 065000, China

**Keywords:** bread wheat, drought tolerance, marker-assisted selection, root system architecture (RSA)

## Abstract

Drought severely threatens wheat production. Under drought conditions, root system architecture (DRSA)-related traits in common wheat significantly affect wheat production. In China, Zhoumai16 is a high-yield winter wheat variety in the Huang-Huai wheat region. It is suitable for high-fertilizer and high-water cultivation and has moderate drought tolerance. DK171 is a newly developed high-yield and stress-tolerant variety, with higher drought tolerance. Thus, identifying genetic loci associated with DRSA-related traits from DK171 and developing available molecular markers are of great importance for enhancing wheat stress tolerance breeding. In this study, DRSA-related traits, including the total root dry weight (DDRW), total root length (DTRL), total root area (DTRA), and the number of root tips (DNRT) under drought stress, were assessed using the hydroponic system in Zhoumai16/DK171 recombinant inbred lines (RIL) population. A total of five quantitative trait loci (QTL) for DRSA-related traits were identified, e.g., *QDDRW.daas-1BL*, *QDTRS.daas-4AL*, *QDNRT.daas-4DS*, *QDTRL.daas-3AL*, and *QDDRW.daas-5D*, and explained 6.1% to 18.9% of the phenotypic variances, respectively. Among these, *QDTRS.daas-4AL* and *QDTRL.daas-3AL* were consistent with previous reports, whereas the *QDDRW.daas-1BL*, *QDNRT.daas-4DS*, and *QDDRW.daas-5D* are novel. The favorable alleles of *QDTRS.daas-4AL* and *QDNRT.daas-4DS* were inherited from Zhoumai16, whereas the favorable alleles for *QDDRW.daas-1BL*, *QDTRL.daas-3AL*, and *QDDRW.daas-5D* were contributed by DK171. Furthermore, five kompetitive allele-specific PCR (KASP) markers, *Kasp_1BL_DTRS* (*QDDRW.daas-1BL*), *Kasp_3AL_DTRS* (*QDTRL.daas-3AL*), *Kasp_4A_DTRS* (*QDTRA.daas-4A*), *Kasp_5D_DDRW* (*QDDRW.daas-5D*), and *Kasp_4D_DNRT* (*QDNRT.daas-4D*), were developed and validated in a diverse panel with 108 wheat varieties mainly from China. Additionally, eight candidate genes related to plant hormone regulation, ABC transporters, and calcium-dependent lipid-binding domain proteins were identified. This study offers new loci, candidate genes, and available KASP markers for wheat drought tolerance breeding and facilitating progress in developing drought-tolerant wheat cultivars.

## 1. Introduction

Common wheat is one of the most crucial staple crops for human consumption and serves as the primary food crop in arid and semi-arid regions worldwide. Drought is the most significant abiotic stress affecting wheat production, severely constraining its sustainable cultivation [1]. Agricultural water inputs are required, resulting in considerable waste of labor and water resources. Therefore, breeding drought-tolerant and water-efficient varieties is the most effective, economical, and environmentally safe approach to reduce the losses caused by drought [2,3,4].

Wheat root system morphology and physiological regulation determine its drought tolerance. Drought tolerance of wheat seedlings is crucial for plant development and production. Root length, surface area, and number of root tips are key root system architecture under drought conditions (DRSA) traits [5,6,7,8]. These characteristics determine the root spatial distribution and primary composition, significantly affecting water and nutrient uptake. Maximum root length forms the basis for water uptake, while the root tip number, root diameter, root length, and root density are closely related to root dry weight, volume, and surface area. Together, these traits determine the plant anchoring strength in the soil profile and its capacity to absorb soil solutions [9,10]. However, the evaluation of these traits is limited by environmental conditions and measurement methods, making it time-consuming and labor-intensive [7,9,10]. Therefore, identifying stable and effective loci for wheat drought tolerance, developing molecular markers for breeding, and elucidating the genetic mechanisms of DRSA-related traits will accelerate the efficient improvement of drought tolerance.

Recently, advancements in high-throughput genotyping technologies, such as re-sequencing and SNP chips [11], effective analysis tools, such as linkage mapping [12,13,14,15,16], and association mapping [17,18] have become common methods for identifying loci of complex traits [6,8,12,13,14]. Over the past two decades, over 50 loci associated with DRSA-related traits have been reported and mainly distributed on chromosomes 1A, 2B, 3A, 3B, 5B, and 6D [6,8,19,20,21]. Although some genetic loci related to root traits in wheat seedlings under drought stress have been reported, many of these loci are linked to SSR markers, which are not efficient for practical use. Additionally, some loci are influenced by complex genetic backgrounds or are linked to non-desirable agronomic traits, making them unsuitable for breeding applications. Therefore, discovering new genetic loci and developing molecular markers that are applicable in breeding holds significant importance for optimizing wheat root systems and achieving high and stable yields. Zhoumai16 (Zhou8425B/Zhou 9) is a high-yield, disease-resistant wheat variety with a significant cultivation area in the Huang-Huai wheat region of China. It is suitable for high-fertilizer and high-water cultivation and has moderate drought tolerance. DK171 (Liangxing66/Shixin828) is a newly developed high-yield and stress-tolerant variety in recent years, with strong drought resistance inherited by Shixin828. In this study, five loci for seedling-stage DRSA-related traits were identified in the Zhoumai16/DK171 recombinant inbred line (RIL) population using the wheat 90K SNP array. The main goal of this study is to uncover the genetic basis of DRSA traits and develop breeding-friendly kompetitive allele-specific PCR (KASP) markers to enhance wheat DRSA-related trait improvement.

## 2. Results

### 2.1. Phenotypic Evaluation

**Figure 1:**
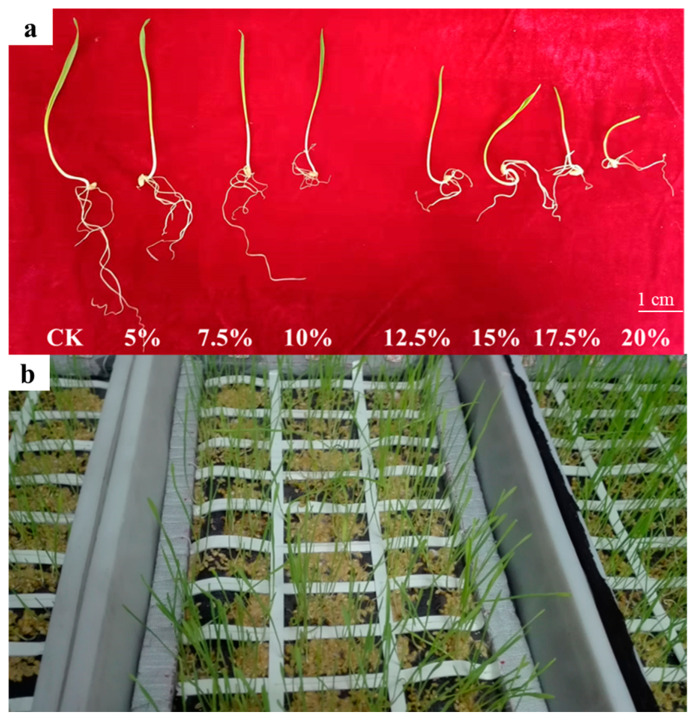
The DRSA-related traits in the Zhoumai16/DK171 RIL population. (**a**) Root growth of wheat seedlings under different concentrations. Root development status under different concentrations of PEG6000, with 12.5% ultimately selected as the drought condition for root phenotypic characterization (5-day). PEG-6000 was used to simulate drought stress. At concentrations of 5–10%, there was no significant reduction in germination potential or germination rate, while concentrations of 12.5–20% significantly inhibited germination. Based on the comparative analysis of relative germination rates and germination potentials at different concentrations, a concentration of 12.5% was selected for the assessment of seedling-related phenotypes. (**b**) Bulk phenotyping of wheat seedling root traits. Bulk evaluation of root phenotypes under 12.5% PEG6000 conditions (8-day growth status).

**Figure A1:**
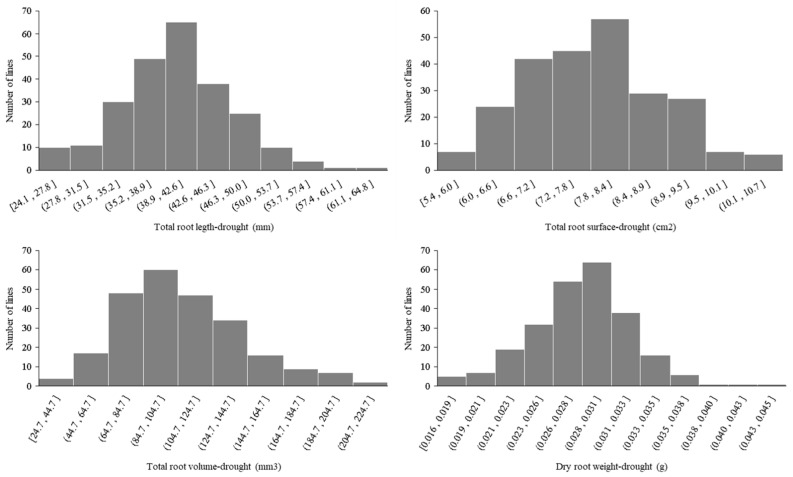
Frequency distribution of the DRSA-related traits in the Zhoumai16/DK171 RIL population.

Zhoumai16 is a high-yielding variety suitable for high-fertility, water-rich regions, while DK171 is a wheat variety that combines high yield with water conservation. For Zhoumai16, the means of total root length (DTRL), total root surface (DTRS), number of root tips (DNRT), and dry root weight (DDRW) were 35.9 cm, 8.0 cm^2^, 123.6, and 0.0312 g, respectively. For DK171, these values were 47.2 cm, 8.9 cm^2^, 168.9, and 0.0339 g. The DTRL, DTRS, DNRT, and DDRW of DK171 were significantly higher than Zhoumai16 (*p* < 0.05). All four DRSA-related traits exhibited continuous and significantly wide variation across the 262 RILs (Table A1 and Figure 1 and Figure A1). The means of DTRL, DTRS, DNRT, and DDRW were 40.2 cm (range: 24.1–63.8 cm), 7.8 cm^2^ (range: 5.4–10.7 cm^2^), 106.9 (range: 24.7–213.7), and 0.0282 g (range: 0.0162–0.0413 g). The standard deviation and coefficient of variation for DTRL, DTRS, DNRT, and DDRW were 6.58 cm (16.4%), 1.06 cm^2^ (13.6%), 35.7 (33.4%), and 0.0041 g (14.5%). Significant correlation was observed between DDRW, DTRL, DTRA, and DNRT, with a correlation coefficient of 0.603 (*p* < 0.05) between DTRL and DTRS (*R*^2^ = 0.56), DTRL and DNRT (*R*^2^ = 0.31), DTRL and DRW (*R*^2^ = 0.32), DTRS and DNRT (*R*^2^ = 0.33), and DNRT and DRW (*R*^2^ = 0.30) (*p* < 0.05).

### 2.2. QTL Identification

This genetic map includes all 21 chromosomes, with red representing QTL and black lines representing backbone SNP markers.

**Figure 2:**
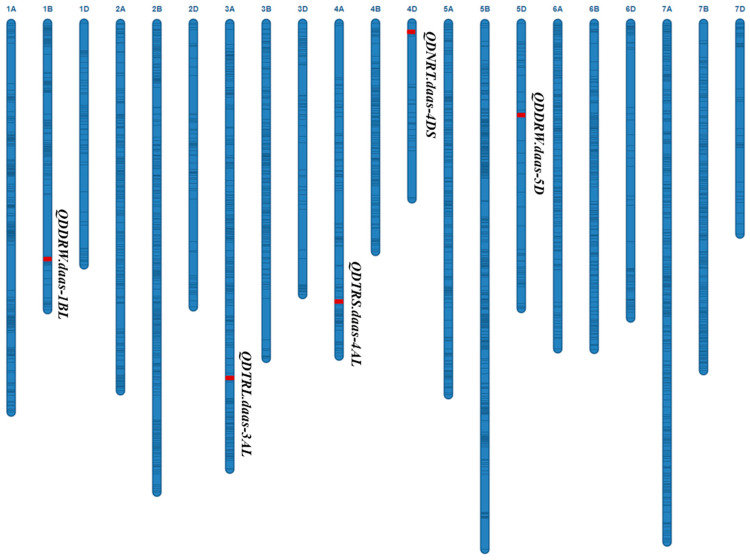
QTL for the DRSA-related traits in the Zhoumai16/DK171 RIL population.

Two QTL for DDRW were identified on chromosomes 1BL and 5DL, referred to as *QDDRW.daas-1BL* (*wsnp_Ex_rep_c67299_65845319*-*Excalibur_rep_c107035_354*) and *QDDRW.daas-5D* (*BobWhite_c5176_1164-RAC875_rep_c78046_324*), respectively. These QTLs explained 18.9% (additive effect: −0.002 g) and 7.5% (additive effect: −0.0007 g) of the total phenotypic variances (PVEs) (Table 1; Figure 1). *QDTRS.daas-4AL* for DTRS was identified on 4AL chromosome (613.6–615.2 Mb, *IAAV7132-wsnp_JD_c38619_27992279*) and explained 9.6% of the PVEs with additive effect −0.275 cm^2^. *QDNRT.daas-4DS* for DNRT was identified on chromosome 4DS (1.2–3.6 Mb, *RAC875_rep_c76650_164-Kukri_c15720_884*) and explained 8.1% of the PVEs with additive effect -7.6. *QDTRL.daas-3AL* for DNRT was identified on chromosome 3AL (650.4–659.4 Mb, *Kukri_rep_c69970_717-Kukri_rep_c103783_1380*) and explained 6.1% (additive effect: −1.272 g) of the PVEs. The favorable allele of *QDTRL.daas-3AL*, *QDDRW.daas-1BL* and *QDDRW.daas-5D* were contributed by DK171, whereas the favorable allele of *QDTRS.daas-4AL* and *QDNRT.daas-4DS* were contributed by Zhoumai16 (Table 1; Figure 2).

### 2.3. Candidate Genes Identification

**Figure 3:**
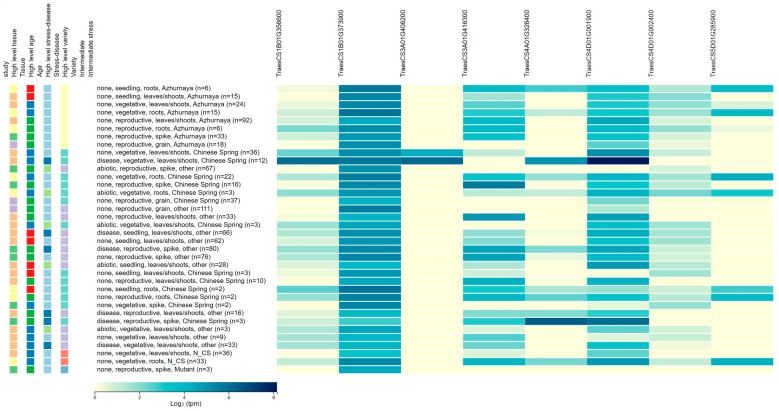
The expression patterns for the eight candidate genes associated with DRSA-related traits. The left side of the figure represents the expression patterns of candidate genes in different tissues and at various developmental stages. The data originates from wheat expression data. The specific transcriptome data can be downloaded from this website (http://wheat-expression.com/, accessed on 24 June 2025).

**Figure 4:**
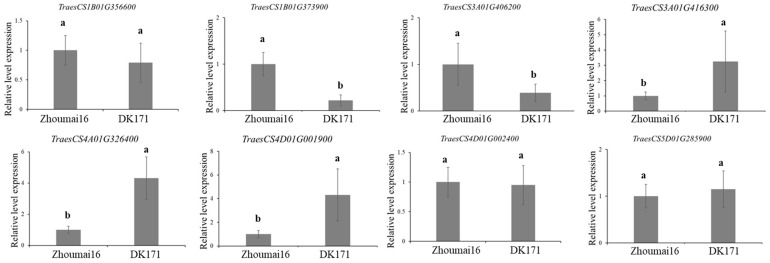
The qRT-PCR results for the candidate genes identified in this study. Transcriptional analysis was conducted in the RIL population of Zhoumai16 and DK171, where different letters indicate significant differences at the *p* < 0.05 level.

Eight candidate genes were identified and involved in the biological metabolism, including the plant hormones, ABC transporter and calcium-dependent lipid-binding domain protein. Two candidate genes for *QDDRW.daas-1BL* were identified, e.g., *TraesCS1B01G356600* and *TraesCS1B01G373900*, encoded the auxin-responsive protein and the ABC transporter family protein, respectively. Both *TraesCS3A01G406200* and *TraesCS3A01G416300* were candidate genes for *QDTRL.daas-3AL* and encoded the gibberellin 20 oxidase and the auxin transport protein, respectively. For *QDTRS.daas-4AL*, *TraesCS4A01G326400* were selected as the candidate gene and encoded the ethylene-responsive transcription factor. *TraesCS4D01G001900* (*QDNRT.daas-4DS*) encoded the calcium-dependent lipid-binding domain protein. *TraesCS4D01G002400* of *QDDRW.daas-5D* encoded the ethylene-responsive transcription factor. *TraesCS5D01G285900* identified at the genetic interval of *QDDRW.daas-5D* and encoded the auxin-induced in root cultures protein (Table 2 and Table A2, Figure 3). The expressions of the seven candidate genes in Zhoumai16 and DK171 were detected using the qRT-PCR. Of these, *TraesCS1B01G356600*, *TraesCS4D01G002400*, and *TraesCS5D01G285900* showed no significant differences between the parents, whereas *TraesCS4D01G002400* and *TraesCS5D01G285900* showed more than 2.3–3.5 folds higher expression in Zhoumai16 compared to DK171; *TraesCS3A01G416300*, *TraesCS4A01G326400*, and *TraesCS4D01G001900* showed more than 3.1–4.3 folds higher expression in DK171 compared to Zhoumai16 (Figure 4).

### 2.4. QTL Validation

All five QTLs were employed in the development of KASP markers. A total of 5 KASP markers, *Kasp-1BL-DDRW* (*QDDRW.daas-1BL, wsnp_Ex_rep_c67299_65845319*, 586.3 Mb) and *Kasp-3AL-DTRL* (*QDTRL.daas-3AL, Kukri_rep_c69970_717*, 650.4 Mb), *Kasp_DTRS_4A* (*QDTRL.daas-4A*, *wsnp_Ex_c7280_12498193*, 725.6 Mb), *Kasp_DDRW_5D* (*QDDRW.daas-5D*, *IACX2960*, 347.5 Mb) and *Kasp_DNRT_4D* (*QDNRT.daas-4D*, *Kukri_rep_c68594_530*, 12.7 Mb), were successfully developed. To validate the efficacy of the 5 KASP markers, a diverse panel of 108 cultivars was employed. For *Kasp-1BL-DDRW*, the allele (AA) account for 60.2% (mean DDRW: 0.0262 g) exhibited lower DDRW compared to the allele (GG), which account for 38.9% with mean DDRW 0.0230 g (*p* < 0.05). For *Kasp-3AL-DTRL*, the allele (AA) account for 66.7% (mean DTRL: 63.52 cm) exhibited higher DTRL compared to the allele (GG), which account for 32.4% with mean DTRL of 56.60 cm^2^ (*p* < 0.05). For *Kasp_DTRS_4A*, the allele (AA) account for 38.9% (mean DTRL: 5.968 cm^2^) exhibited lower DTRS compared to the allele (GG), which account for 55.6% with mean DTRS 6.993 cm^2^ (*p* < 0.05). For *Kasp_DDRW_5D*, the favorable allele (CC 10.2%, a mean DRW of 0.0297 g) showed higher DDRW than unfavorable allele (TT, 88.9%, mean DDRW of 0.0244 g) at *p* = 0.05 level. For *Kasp_DNRT_4D*, the favorable allele (CC 65.7%, mean DNRT of 97.8) showed higher DNRT than the unfavorable allele (TT, 30.6%, mean DNRT of 83.0) (*p* = 0.05) (Table 3, Table 4 and Table A3).

To assess the accuracy of markers in detecting root phenotypes in natural populations, we selected the median value as the threshold for each trait. Phenotype values below the median were categorized as non-superior phenotypes, while those above were classified as superior phenotypes. We calculated the consistency rate between genotypes and phenotypes to provide a reference for the detection and evaluation of wheat root systems under drought conditions. The accuracy rates of *Kasp_1BL_DTRS*, *Kasp_3AL_DTRS*, *Kasp_DTRS_4A*, *Kasp_DDRW_5D*, and *Kasp_DNRT_4D* in detecting superior and non-superior phenotypes for DTRS, DDRW, and DNRT were 66.0% and 75.5%; 90.6% and 92.5%; 60.4% and 73.6%; 58.5% and 79.2%; 52.8% and 66.0%, respectively.

## 3. Discussion

Roots are the main parts of plants that take up water and nutrients. They also provide support and stability. Wheat has a fibrous root system. As it grows after germination, it develops adventitious roots. These roots are vital for anchoring the plant and absorbing water and nutrients [2,13]. They form the genetic foundation for desirable traits like drought tolerance, salt tolerance, and resistance to falling over (lodging). However, studying the root system of mature plants is difficult. Soil conditions and farming practices easily affect it. Traditional methods for taking root samples are often destructive and complicated, making it hard to measure root traits efficiently [5,14]. Research shows that the root systems of young seedlings are different. They are strongly inherited and less affected by the environment. These young roots can indicate the shape and spread of the mature root system and are closely linked to the plant’s ability to handle stress. A good root structure is the basis for having enough root volume and surface area to function well.

To adapt to diverse environments, wheat varieties have accumulated numerous genetic variations. Genetic enhancement of crop roots has seldom been analyzed [14,15]. The identification of QTL provides an effective strategy to develop molecular markers and identify candidate genes [16]. A thorough understanding of the genetic foundation of drought root system architecture traits would aid in optimizing root systems under conditions of nutrient deficiency [2,3,5,6,7,8,12,13,14]. Several QTLs associated with root system architecture-related traits have been uncovered in wheat [17,18]. Root system architecture is a highly adaptable trait across various environments [22]; QTLs governing root system architecture under drought conditions are crucial for enhancing drought tolerance. Over 10 loci influencing the number of root tips under drought stress have been mapped on chromosomes 4A, 4B, 4D, 5B, 1D, 6D, and 7D [23]. The locus on chromosome 4D (360.3–396.8 Mb) is different from the loci identified in this study (*QDNRT.daas-4DS* 1.2–3.6 Mb). Thus, *QDNRT.daas-4DS* is a new loci. Over 10 loci for root surface area under drought stress were identified on chromosomes 1D, 2A, 2B, 2D, 3B, 4A, 5B, 5D, 7A, and 7D in common wheat [23]. Of these, the locus on 4A (565.6–598.2 Mb) is nearly adjacent to the *QDTRS.daas-4AL* (613.6–615.2 Mb).

In total, 12 loci for total root length under drought stress were identified on chromosomes 1A, 1D, 2A, 2D, 3A, 3B, 3D, 4B, 5B, 5D, 7A, and 7D [23,24,25]. In this study, we have identified a locus for total root length under drought stress, *QDTRL.daas-3AL* (650.4–659.4 Mb), which is near the loci on 3AL (632.8–646.3 Mb). Until now, eight loci for DDRW were identified on chromosomes 1B, 2A, 2D, 4A, 4B, 5A, 7A, and 7D [1,26,27,28,29]. We identified two loci for DDRW, *QDDRW.daas-1BL* (586.3–609.0 Mb) and *QDDRW.daas-5D* (378.9–393.5 Mb), which differ from the loci located on chromosome 1B (120.2–159.6 Mb) and 5D (489.6–540.3 Mb) mentioned above. Thus, both *QDDRW.daas-1BL* and *QDDRW.daas-5D* are novel.

The genes associated with plant height and vernalization may also have significant effects on root system architecture-related traits [30]. Over the past 80 years, several genetic loci associated with plant height and vernalization have been identified in common wheat, and a number of functional genes have been cloned on chromosome 1B (*Rht2*/*Rht10* at 19.18 Mb), 4D (*SVP3-4D/BM1-4D* at 469.46 Mb, *Vrn2-4D/ZCCT1-4D* at 509.43 Mb), and 5D (*TaDEP1-5D* at 329.11 Mb, *Rht23* at 524.96 Mb, and *Vrn1-5D* at 470.00 Mb) [31], and 6A (Rht24, 411.93–414.88 Mb) [32]. Based on physical positions, the loci *QDTRS.daas-4AL* (613.6–615.2 Mb) and *QDNRT.daas-4DS* (1.2–3.6 Mb), *QDTRL.daas-3AL* (650.4–659.4 Mb), *QDDRW.daas-1BL* (586.3–609.0 Mb), and *QDDRW.daas-5D* (378.9–393.5 Mb) are different from the reported plant height and vernalization genes.

Notably, compared with previous results and meta-analyses, *QDDRW.daas-1BL*, *QDNRT.daas-4DS*, and *QDDRW.daas-5D* were novel. We have presented the linkage mapping results for agronomic traits in the Zhoumai16/DK171 RIL population [15] and pinpointed several genomic regions linked to both drought root system architecture-related traits and agronomic traits. Specifically, *QDDRW.daas-1BL* (586.3–609.0 Mb) overlaps with a QTL cluster (556–654 Mb) influencing kernel number per spike (KNS), PH, and flag leaf width (FLW), and *QDDRW.daas-5D* (378.9–393.5 Mb) co-locates with a QTL cluster (277.0–491.2 Mb) related to KNS, FLW, TKW, and heading date [15]. These findings suggest that the loci associated with drought response and survival mechanisms may also serve as targets for enhancing yield potential and stability.

The limitation raised regarding the absence of control experiments in this study, which currently prevents definitive verification of whether the identified QTLs are specific to drought conditions. Although these QTLs demonstrated significant effects under drought stress, their potential functionality under non-stress conditions remains unexamined. Unfortunately, insufficient seed availability precluded the inclusion of control treatments in the present experiment. To address this gap, subsequent phenotyping of root-related traits will be conducted under optimal growing conditions. The acquired data will support two analytical approaches: direct QTL mapping under control conditions to compare with drought-induced QTLs, and mapping based on trait ratios between stress and control conditions. These analyses will help distinguish QTLs unique to drought response from those constitutive to plant growth—a distinction critical for breeding applications. While the current study provides directly relevant insights for drought tolerance breeding and genetic dissection under water-limited environments, further validation under controlled conditions will significantly enhance the biological interpretation and practical utility of these loci.

A total of eight candidate genes were identified, primarily implicated in the biological metabolism of plant hormones and calcium-dependent lipid-binding domain proteins. Among these, *TraesCS4D01G001900* (*QDNRT.daas-4DS*) encodes a CDPK-related kinase, playing a crucial role in diverse signaling pathways for root growth and development [33,34], such as root hair growth and cell length [35]. Additionally, *TraesCS1B01G373900*, associated with *QDDRW.daas-1BL*, encodes an ABC transporter family protein essential for primary root growth and shoot development. Root development is governed by various plant hormones [36]. *TraesCS4A01G326400* (*QDTRS.daas-4AL*) and *TraesCS4D01G002400* (*QDNRT.daas-4DS*) encode ethylene-responsive transcription factors [37]. Ethylene plays diverse roles in growth, development, signal transduction, and cell differentiation, including root growth [8,9], and influences drought root system architecture-related traits like root hair and cluster root formation [25]. *TraesCS1B01G356600* (*QDDRW.daas-1BL*), *TraesCS4D01G002400* (*QDNRT.daas-4DS*), and *TraesCS5D01G285900* (*QDDRW.daas-5D*) encode the auxin-responsive proteins, auxin transport protein, and auxin-induced protein 12 in root cultures. Auxin, a core regulator, integrates with other plant hormones to regulate root development. The biosynthesis, transport, and signaling pathways of auxin, particularly indole-3-acetic acid, are crucial for plant root development [20]. *TraesCS3A01G406200* (*QDTRL.daas-3AL*) encodes the gibberellin 20 oxidase 2 (GA20ox), a key enzyme in gibberellin synthesis [36,37], which regulate various stages of plant growth and development, promoting seed germination, plant growth, flowering induction, and other biological functions.

In this study, the candidate genes were preliminarily screened through bioinformatic annotation and expression profiling analyses. These candidate genes currently serve only as reference targets as their biological functions remain to be experimentally validated. To systematically characterize these candidate genes, the following research pipeline were applied: (1) construction of a secondary mapping population coupled with KASP marker development for high-resolution genetic mapping; (2) comprehensive identification of target genes through integrated transcriptomic and genomic variation analyses; (3) functional validation employing both gene editing (e.g., CRISPR/Cas9) and transgenic complementation approaches. It should be emphasized that the KASP markers utilized in this study were specifically designed as genetic linkage markers rather than functional markers.

Traditional wheat breeding primarily focuses on yield and disease-related traits, with root system architecture closely linked to yield traits. Although traditional breeding has improved root system characteristics, the selection process remains lengthy and less efficient due to the challenges in field measurement of drought root system architecture-related traits [19]. Moreover, seedling root development is crucial for early wheat growth. KASP markers have been widely adopted for detecting genetic variations in wheat, enabling high-throughput genotyping. By utilizing genotype data from wheat SNP arrays for QTL mapping and genome-wide association studies, linked SNPs can be converted into KASP markers, which can then be directly applied in marker-assisted selection breeding programs. This approach facilitates the efficient identification and selection of desirable traits in wheat breeding efforts [19]. KASP markers are extensively applied in the improvement of yield, disease resistance, and quality traits in wheat. In this study, we successfully developed based on tightly linked SNP markers. The accuracy rates of *Kasp_1BL_DTRS*, *Kasp_3AL_DTRS*, *Kasp_DTRS_4A*, *Kasp_DDRW_5D*, and *Kasp_DNRT_4D* in detecting superior and non-superior phenotypes for DTRS, DDRW, and DNRT were 66.0% and 75.5%; 90.6% and 92.5%; 60.4% and 73.6%; 58.5% and 79.2%; 52.8% and 66.0%, respectively. Thus, these KASP markers could be used as valuable tools in MAS breeding programs. Additionally, accessions carrying more favorable alleles and exhibiting superior DRSA traits along with desirable agronomic characteristics, such as Jinmai 61, Liangxing 99, Yumai 35, Yumai 47, Liangxing 66, Bainong 64, Lumai 8, Yanzhan 4110, Zhengmai 366, Jimai 22, and Aikang 58, are recommended as parental lines for the improvement of drought root system architecture traits.

## 4. Materials and Methods

### 4.1. Plant Materials and Phenotypic Traits

Zhoumai16 is a high-yield winter wheat variety in the Huang-Huai wheat region. It is suitable for high-fertilizer and high-water cultivation and has moderate drought tolerance. DK171 is a newly developed high-yield and stress-tolerant winter wheat variety, with higher drought tolerance. This study utilized a Zhoumai16/DK171 F_2:6_~ RIL population to conduct hydroponic experiments under greenhouse drought stress conditions, measuring DRSA-related traits with three replicates.

The standard Hoagland nutrient solution includes: Macronutrients (mg/L): Calcium Nitrate (Ca(NO_3_)_2_·4H_2_O) at 945 mg/L, Potassium Nitrate (KNO_3_) at 607 mg/L, Ammonium Dihydrogen Phosphate (NH_4_H_2_PO_4_) at 115 mg/L, Magnesium Sulfate (MgSO_4_·7H_2_O) at 493 mg/L. Micronutrients (mg/L): EDTA-Iron (Fe-EDTA) at 20 mg/L, Boric Acid (H_3_BO_3_) at 2.86 mg/L (or Borax), Manganese Sulfate (MnSO_4_·H_2_O) at 2.13 mg/L, Zinc Sulfate (ZnSO_4_·7H_2_O) at 0.22 mg/L, Copper Sulfate (CuSO_4_·5H_2_O) at 0.08 mg/L, and Ammonium Molybdate ((NH_4_)_6_Mo_7_O_24_·4H_2_O) at 0.02 mg/L. The solution pH should be adjusted to 5.5–6.5 using acid/base before use. Prepare 1× full-strength Hoagland solution in advance and adjust its pH to 5.8–6.0. Weigh 5.0 g, 7.5 g, 10.0 g, 12.5 g, 15.0 g, 17.5 g, and 20.0 g of PEG 6000 into seven clean beakers. Add about 60 mL of the pre-warmed (50–60 °C) 1× Hoagland solution to each beaker and stir gently on a magnetic stirrer until the PEG 6000 is completely dissolved. Transfer each solution to its corresponding 100 mL volumetric flask, bring the volume to the mark with additional 1× Hoagland solution, and mix thoroughly by inverting the flasks.

The root dry weights of Zhoumai16 plants subjected to 5%, 7.5%, 10%, 12.5%, 15%, 17.5%, and 20% PEG6000 were 0.0128 g, 0.0122 g, 0.0098 g, 0.0090 g, 0.0065 g, 0.0060 g, 0.0052 g, and 0.0026 g, respectively. A 12.5% concentration of PEG 6000 was added to the culture medium to simulate drought conditions. The methodology is as follows: 20 wheat seeds from each line were randomly selected and surface-sterilized with 10% H_2_O_2_ for 20 min, then placed in Petri dishes containing moist filter paper. When the coleoptile length reached approximately 2 cm, the seedlings were transferred to plastic trays (53 × 27 cm) with Hoagland nutrient solution supplemented with 12.5% PEG 6000 to induce osmotic stress.

These trays were then placed in a constant temperature culture room maintained at 25 °C, with 16 h light and 8 h darkness. After three weeks of growth in the greenhouse, seedling DRSA-related traits, including DDRW, DTRL, DTRA, and DNRT, were measured using the WinRHIZO software V1.0 (https://www.quantitative-plant.org/software/winrhizo, accessed on 24 June 2025) (Regent, Vancouver, BC, Canada). The specific method was as follows: thoroughly washed wheat roots were neatly arranged in a scanning tray, and scanned images were obtained using an Expression 11000XL scanner (Seiko Epson Corporation, Nagano Prefecture, Japan). The scanned images were analyzed using WinRHIZO software. Five plants were measured for each line, with three biological replicates.

This study used 108 varieties to validate the effects of KASP markers. The phenotypic values of DDRW, DTRL, DTRA, and DNRT for this validation population were also uniformly identified using the aforementioned method.

### 4.2. Genome Wide Linkage Mapping

The Zhoumai16/DK171 RIL population was genotyped using the wheat 90K SNP chip (CapitalBio Corporation, Beijing, China). The quality control followed: SNPs were set at missing data exceeding 20% or minor allele frequency (MAF) below 0.5. Filtered SNPs were classified into bin markers by IciMapping v4.2 [17]. Subsequently, the regression mapping algorithm in JoinMap v4.0 was used to calculate linkage distances for the obtained BIN markers and construct a higher-density linkage map. The successfully constructed linkage map has been previously reported by Wen et al. [18] and Li et al. [15]. Based on the constructed high-density SNP genetic map and the obtained root DRSA-related traits under drought, genome-wide linkage mapping was conducted by the inclusive composite interval mapping (ICIM-add) using IciMapping v4.1 [17]. The logarithm of odds (LOD) threshold for significant QTLs was determined to be 2.60 based on 1000 permutations. The physical positions were determined by IWGSC v1.0.

### 4.3. Identification of Candidate Genes for Drought-Related Traits

To identify the candidate genes associated with drought-related trait QTLs detected in the Zhoumai16/DK171 RIL population, high-confidence annotation genes within the LD block region surrounding each QTL peak SNP were extracted from the wheat IWGSC v1.0 [21]. Combining annotation, high-confidence genes with relevant annotated functions and differences in the coding regions between the two parents were screened as candidate genes. To investigate the expression patterns and identify candidate genes with notable transcription levels in seedlings or root tissues, we utilized the publicly accessible *Triticum aestivum* gene expression database [12] (http://wheat-expression.com/, accessed on 24 June 2025). After phenotyping, roots were sampled, and RNA was extracted from the root samples using the TRIzol method. cDNA was synthesized using the HiScript II cDNA Synthesis Kit. Primers for qRT-PCR were designed with Primer Premier 5.0. The reaction mixture consisted of 20 µL, including 2 µL of cDNA, 10 µL of ChamQ Universal SYBR qPCR Master Mix, and 0.4 µL of each primer. *TaActin*1 was used as an internal control to normalize the expression levels of different samples. The gene expression levels were analyzed using the 2^−ΔΔCT^ method. All assays were performed with two biological replicates and three technical replicates.

### 4.4. KASP Marker Development and Validation

For all loci, flanking SNPs were converted to KASP markers [19], designed using PolyMarker (http://www.polymarker.info/, accessed on 24 June 2025). The 384-well plates were analyzed on a PHERAstarplus SNP, and genotyping was conducted by KlusterCaller (LGC) (https://www.lgcstandards.com/, accessed on 24 June 2025) (London, UK). All developed KASP markers required genetic effect validation using 108 varieties mainly from the Yellow and Huai Wheat Region [20]. These 108 materials primarily include the main popularized varieties, key backbone parents, and representative lines from the Yellow and Huai River Valleys Wheat Zone.

## 5. Conclusions

In conclusion, this study highlights the critical role of drought-related root system architecture in enhancing wheat resilience to drought. By analyzing the Zhoumai16/DK171 RIL population, five QTLs associated with DRSA-related traits were identified, including novel loci *QDDRW.daas-1BL, QDNRT.daas-4DS*, and *QDDRW.daas-5D*. These QTLs explained 6.1% to 18.9% of the phenotypic variances, with favorable alleles contributed by both Zhoumai16 and DK171. Additionally, five KASP markers were developed and validated, providing valuable tools for MAS breeding. The identification of eight candidate genes further enriches the genetic resources for wheat drought tolerance. This research advances our understanding of the genetic basis of drought resistance and offers practical tools for breeding drought-tolerant wheat cultivars.

## Figures and Tables

**Table 1:** QTL for DRSA-related traits in Zhoumai16/DK171 RIL population.

QTL	Chromosome	Genetic Interval	Physical Position (Mb)	LOD	R^2^	Add
*QDDRW.daas-1BL*	1B	*wsnp_Ex_rep_c67299_65845319~* *Excalibur_rep_c107035_354*	586.3–609.0	11.1	18.9	−0.002
*QDTRL.daas-3AL*	3A	*Kukri_rep_c69970_717~Kukri_rep_c103783_1380*	650.4–659.4	3.0	6.1	−1.27
*QDTRS.daas-4AL*	4A	*IAAV7132~wsnp_JD_c38619_27992279*	613.6–615.2	3.5	9.6	0.28
*QDNRT.daas-4DS*	4D	*RAC875_rep_c76650_164~Kukri_c15720_884*	1.2–3.6	3.2	8.1	7.61
*QDDRW.daas-5D*	5D	*BobWhite_c5176_1164~RAC875_rep_c78046_324*	378.9–393.5	3.1	7.5	−0.001

**Table 2:** The candidate genes for DRSA-related traits identified in the Zhoumai16/DK171 RIL population.

QTL	Candidate Gene	Chromosome	Start (bp)	Annotation
*QDDRW.daas-1BL*	*TraesCS1B01G356600*	1B	585539303	Auxin-responsive protein
*QDDRW.daas-1BL*	*TraesCS1B01G373900*	1B	604319205	ABC transporter A family protein
*QDTRL.daas-3AL*	*TraesCS3A01G406200*	3A	650429434	Gibberellin 20 oxidase 2
*QDTRL.daas-3AL*	*TraesCS3A01G416300*	3A	658983005	Auxin transport protein (BIG)
*QDTRS.daas-4AL*	*TraesCS4A01G326400*	4A	613543523	Ethylene-responsive transcription factor
*QDNRT.daas-4DS*	*TraesCS4D01G001900*	4D	1239483	Calcium-dependent lipid-binding domain protein
*QDNRT.daas-4DS*	*TraesCS4D01G002400*	4D	1288444	Ethylene-responsive transcription factor
*QDDRW.daas-5D*	*TraesCS5D01G285900*	5D	386209788	Auxin-induced in root cultures protein 12

**Table 3:** Effects of *Kasp_4AL_DTRS*, *Kasp_4DS_DNRT*, and *Kasp_5D_DDRW* on DRSA-related traits in the natural population.

Marker Name	QTL	Genotype ^a^	Number of Lines	Phenotype	*p*-Value
*Kasp_1BL_DDRW*	*QDDRW.daas-1BL*	*AA*	74	6.80 (DTRS)	0.023 *
		GG	33	5.85 (DTRS)	
*Kasp_3AL_DTRL*	*QDTRL.daas-3AL*	*AA*	72	63.52 (DTRL)	0.010 *
		GG	35	56.60 (DTRL)	
*Kasp_4AL_DTRS*	*QDTRS.daas-4AL*	*GG*	60	6.99 (DTRS)	0.021 *
		AA	42	5.97 (DTRS)	
*Kasp_4DS_DNRT*	*QDNRT.daas-4DS*	*TT*	33	83.0 (DNRT)	0.009 *
		CC	71	97.7 (DNRT)	
*Kasp_5D_DDRW*	*QDDRW.daas-5D*	*CC*	11	0.0297 (DDRW)	0.039 *
		TT	96	0.0245 (DDRW)	

^a^ The italic is the favorable allele. * Significant at *p* < 0.05.

**Table 4:** The primers of the KASP markers identified in this study.

Kasp Name	Primer	Sequence
*Kasp-1BL-DDRW*	FAM	GAAGGTGACCAAGTTCATGCTTTGAGGCGACCACCCTGA
	HEX	GAAGGTCGGAGTCAACGGATTTTGAGGCGACCACCCTGG
	COMMON	GCAGCCGTTATTCAACTTCTAA
*Kasp-3AL-DTRL*	FAM	GAAGGTGACCAAGTTCATGCTCCTTCTGGATTGATGGTTCTCA
	HEX	GAAGGTCGGAGTCAACGGATTCCTTCTGGATTGATGGTTCTCG
	COMMON	TCTGCCTCGAAGTCTTCATTT
*Kasp_4AL_DTRS*	FAM	GAAGGTGACCAAGTTCATGCTCATTGCCAAATGTTTGCTGTATT
	HEX	GAAGGTCGGAGTCAACGGATTCATTGCCAAATGTTTGCTGTATC
	COMMON	CATTATCAGATGATACCACGTCG
*Kasp_4DS_DNRT*	FAM	GAAGGTGACCAAGTTCATGCTTGAACTCGGCTGATACCAGA
	HEX	GAAGGTCGGAGTCAACGGATTGAACTCGGCTGATACCAGG
	COMMON	GGTGATGGCGAACCTAGAAAC
*Kasp_5D_DDRW*	FAM	GAAGGTGACCAAGTTCATGCTCATTGCCAAATGTTTGCTGTATT
	HEX	GAAGGTCGGAGTCAACGGATTCATTGCCAAATGTTTGCTGTATC
	COMMON	CATTATCAGATGATACCACGTCG

The underlined nucleotides are the FAM and HEX primers.

**Figure A2:**
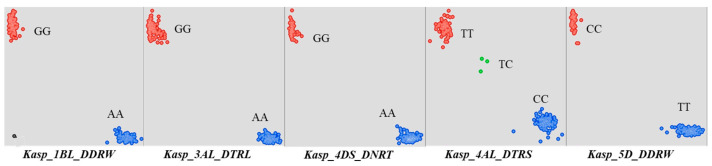
The genotype of the KASP markers.

## Data Availability

All datasets generated for this study are included in the article; further inquiries can be directed to the first author.

## Appendix A

**Table A1:** The drought root system architecture-related traits in Zhoumai16/DK171 RIL population.

Accessions	Total Root Length-Drought (mm)	Total Root Surface-Drought (cm^2^)	Total Root Volume-Drought (mm^3^)	Dry Root Weight-Drought (g)
LINE1	26.5	5.8	201.0	0.0203
LINE2	25.8	6.0	24.7	0.0169
LINE3	24.1	5.4	35.7	0.0181
LINE4	27.6	6.5	48.3	0.0277
LINE5	35.0	7.6	45.3	0.0276
LINE6	42.5	8.6	73.3	0.0254
LINE7	35.5	8.7	94.3	0.0295
LINE8	38.1	6.1	81.0	0.0271
LINE9	35.5	6.9	105.7	0.0213
LINE10	50.0	10.3	79.3	0.0268
LINE11	26.8	5.7	108.0	0.0235
LINE12	24.6	6.5	57.3	0.0176
LINE13	35.9	8.1	48.7	0.0267
LINE14	37.9	6.4	63.0	0.0209
LINE15	60.6	8.5	70.7	0.0244
LINE16	40.5	9.2	136.7	0.0237
LINE17	42.5	6.7	99.0	0.0279
LINE18	36.2	7.2	137.3	0.0248
LINE19	31.9	6.2	86.0	0.0246
LINE20	NN	NN	NN	NN
LINE21	49.1	8.1	79.3	0.0232
LINE22	42.8	7.9	119.7	0.0284
LINE23	41.8	7.4	104.7	0.0305
LINE24	40.8	6.1	147.0	0.0233
LINE25	44.1	7.8	99.7	0.0286
LINE26	40.6	8.1	84.7	0.0237
LINE27	45.3	8.3	90.0	0.0226
LINE28	43.6	10.7	85.7	0.0271
LINE29	39.4	8.4	103.3	0.0278
LINE30	46.7	8.9	140.0	0.0255
LINE31	NN	NN	NN	NN
LINE32	41.9	8.3	128.3	0.0273
LINE33	35.2	7.6	198.7	0.0310
LINE34	34.5	7.0	96.7	0.0315
LINE35	41.6	8.2	116.7	0.0231
LINE36	38.0	7.2	156.7	0.0206
LINE37	37.4	6.9	84.0	0.0342
LINE38	40.5	10.4	99.0	0.0235
LINE39	35.9	6.0	129.3	0.0298
LINE40	39.3	8.2	82.3	0.0346
LINE41	35.0	8.6	87.0	0.0272
LINE42	40.7	8.3	91.0	0.0259
LINE43	37.0	7.4	163.0	0.0246
LINE44	47.5	9.0	105.0	0.0285
LINE45		6.8	103.7	0.0297
LINE46	40.4	7.7	124.7	0.0292
LINE47	47.3	7.9	101.7	0.0313
LINE48	35.9	9.9	134.3	0.0331
LINE49	35.0	6.0	88.0	0.0238
LINE50	48.5	9.8	114.0	0.0284
LINE51	36.1	8.4	117.0	0.0273
LINE52	52.8	7.6	79.0	0.0296
LINE53	36.2	7.8	114.0	0.0304
LINE54	56.6	10.3	72.0	0.0298
LINE55	42.5	8.1	176.3	0.0285
LINE56	41.9	8.2	128.0	0.0261
LINE57	35.7	7.1	113.3	0.0275
LINE58	36.9	6.9	96.0	0.0303
LINE59	48.0	7.5	126.0	0.0332
LINE60	32.2	7.8	116.3	0.0278
LINE61	47.5	8.6	77.3	0.0266
LINE62	40.3	8.1	129.0	0.0245
LINE63	37.1	7.1	99.0	0.0267
LINE64	44.5	7.5	95.7	0.0299
LINE65	47.8	6.9	139.0	0.0310
LINE66	40.1	7.0	117.7	0.0364
LINE67	41.2	9.2	122.3	0.0295
LINE68	42.8	7.7	141.0	0.0288
LINE69	39.7	6.2	151.7	0.0304
LINE70	NN	NN	NN	NN
LINE71	37.1	9.2	114.7	0.0278
LINE72	41.9	7.8	109.0	0.0315
LINE73	38.6	7.3	112.7	0.0289
LINE74	47.3	9.0	95.0	0.0370
LINE75	42.1	10.6	122.0	0.0221
LINE76	37.1	6.8	144.3	0.0374
LINE77	NN	NN	NN	NN
LINE78	39.0	7.8	89.7	0.0274
LINE79	39.6	6.9	109.7	0.0301
LINE80	43.8	8.5	135.0	0.0255
LINE81	43.1	8.4	102.7	0.0276
LINE82	45.0	7.1	100.0	0.0283
LINE83	40.3	7.8	123.3	0.0256
LINE84	30.8	6.8	82.0	0.0317
LINE85	34.2	7.8	62.3	0.0278
LINE86	NN	NN	NN	NN
LINE87	41.1	6.2	93.0	0.0282
LINE88	37.3	9.5	136.3	0.0236
LINE89	50.6	9.1	143.3	0.0286
LINE90	29.8	6.4	98.3	0.0271
LINE91	30.4	8.7	96.7	0.0274
LINE92	45.5	9.3	100.7	0.0308
LINE93	38.1	7.0	114.0	0.0342
LINE94	44.4	7.9	141.7	0.0282
LINE95	46.9	8.7	139.0	0.0363
LINE96	38.1	7.4	153.3	0.0301
LINE97	27.7	6.8	99.7	0.0247
LINE98	32.8	6.4	53.3	0.0245
LINE99	34.9	8.2	156.3	0.0309
LINE100	46.1	8.0	99.7	0.0298
LINE101	42.4	7.4	117.3	0.0323
LINE102	48.4	8.1	68.3	0.0345
LINE103	50.4	9.2	117.0	0.0272
LINE104	47.3	8.6	121.3	0.0431
LINE105	40.3	7.7	164.7	0.0297
LINE106	45.8	6.0	80.0	0.0305
LINE107	35.7	7.0	145.0	0.0329
LINE108	50.4	8.1	87.7	0.0296
LINE109	43.2	8.2	136.3	0.0287
LINE110	NN	NN	NN	0.0286
LINE111	41.0	6.4	71.7	0.0307
LINE112	NN	NN	NN	0.0274
LINE113	NN	NN	NN	0.0284
LINE114	NN	NN	NN	0.0413
LINE115	NN	NN	NN	0.0302
LINE116	54.5	10.3	97.0	0.0320
LINE117	44.6	7.7	109.7	0.0328
LINE118	NN	NN	NN	0.0256
LINE119	NN	NN	NN	0.0312
LINE120	41.3	7.0	104.3	0.0272
LINE121	38.3	6.0	91.0	0.0278
LINE122	50.3	7.6	77.7	0.0325
LINE123	45.1	8.3	165.3	0.0317
LINE124	44.7	8.2	77.0	0.0306
LINE125	37.8	7.2	101.7	0.0328
LINE126	38.2	6.2	117.0	0.0227
LINE127	30.7	5.7	164.7	0.0206
LINE128	43.8	7.9	55.0	0.0288
LINE129	57.4	7.7	102.7	0.0327
LINE130	49.5	7.8	147.7	0.0345
LINE131	44.4	7.6	125.3	0.0354
LINE132	38.6	7.3	145.3	0.0292
LINE133	45.8	8.6	97.7	0.0278
LINE134	44.3	9.0	67.3	0.0286
LINE135	36.1	6.7	117.0	0.0296
LINE136	34.8	7.3	126.3	0.0322
LINE137	42.3	8.2	143.3	0.0294
LINE138	27.3	6.3	151.3	0.0264
LINE139	36.2	7.5	68.0	0.0308
LINE140	42.1	7.9	137.0	0.0299
LINE141	NN	NN	NN	NN
LINE142	50.2	8.3	204.0	0.0304
LINE143	28.0	6.7	98.7	0.0162
LINE144	36.1	7.1	66.3	0.0284
LINE145	32.9	6.4	70.0	0.0258
LINE146	38.6	9.3	83.3	0.0221
LINE147	45.2	8.6	88.7	0.0280
LINE148	44.6	8.9	196.7	0.0278
LINE149	40.5	8.2	187.3	0.0326
LINE150	46.6	9.4	126.7	0.0308
LINE151	45.4	7.7	150.7	0.0287
LINE152	41.3	8.8	115.3	0.0255
LINE153	37.4	6.8	113.3	0.0251
LINE154	41.0	7.4	89.3	0.0330
LINE155	39.5	6.2	108.0	0.0243
LINE156	56.6	7.9	127.3	0.0207
LINE157	30.6	7.0	142.0	0.0236
LINE158	41.0	9.0	48.0	0.0318
LINE159	49.9	9.9	165.3	0.0323
LINE160	46.7	7.4	213.7	0.0372
LINE161	46.6	8.7	87.7	0.0268
LINE162	33.0	7.0	115.0	0.0293
LINE163	39.9	8.5	86.0	0.0242
LINE164	37.1	7.1	67.7	0.0267
LINE165	43.7	8.6	60.3	0.0350
LINE166	44.4	8.6	119.3	0.0293
LINE167	63.8	9.2	145.0	0.0306
LINE168	41.0	7.6	136.0	0.0279
LINE169	39.0	9.0	122.7	0.0287
LINE170	35.7	7.7	180.0	0.0241
LINE171	52.4	9.0	59.7	0.0230
LINE172	49.4	10.1	172.7	0.0343
LINE173	41.9	7.2	142.3	0.0279
LINE174	44.7	9.2	101.7	0.0271
LINE175	36.8	8.6	121.0	0.0268
LINE176	49.0	8.6	81.0	0.0346
LINE177	48.7	8.8	131.3	0.0304
LINE178	45.5	9.1	202.0	0.0301
LINE179	44.1	8.2	85.7	0.0301
LINE180	32.6	6.7	86.7	0.0253
LINE181	33.7	7.8	53.0	0.0259
LINE182	33.8	6.4	72.0	0.0230
LINE183	50.8	9.7	94.0	0.0212
LINE184	40.6	7.6	71.3	0.0324
LINE185	40.5	8.9	107.0	0.0257
LINE186	41.9	8.2	109.3	0.0277
LINE187	45.8	9.3	96.3	0.0314
LINE188	34.6	7.0	138.0	0.0316
LINE189	35.0	8.0	114.0	0.0317
LINE190	32.0	7.3	120.0	0.0203
LINE191	35.3	7.1	51.3	0.0314
LINE192	41.6	7.2	116.7	0.0311
LINE193	43.9	7.7	58.0	0.0369
LINE194	44.7	8.5	124.0	0.0280
LINE195	42.2	9.3	117.3	0.0323
LINE196	39.1	7.3	189.3	0.0292
LINE197	NN	NN	NN	NN
LINE198	43.5	7.9	85.7	0.0321
LINE199	29.3	7.1	130.0	0.0195
LINE200	51.9	8.3	77.3	0.0233
LINE201	39.5	8.5	65.7	0.0246
LINE202	40.1	7.1	44.7	0.0212
LINE203	40.3	8.2	87.7	0.0284
LINE204	38.6	8.3	96.7	0.0217
LINE205	39.2	8.5	65.0	0.0263
LINE206	39.2	8.2	82.7	0.0229
LINE207	49.0	7.9	72.7	0.0232
LINE208	35.8	7.4	102.0	0.0221
LINE209	NN	NN	NN	NN
LINE210	39.1	7.3	56.7	0.0234
LINE211	44.0	7.9	68.3	0.0306
LINE212	NN	NN	NN	NN
LINE213	34.0	7.8	133.7	0.0309
LINE214	51.4	7.2	87.0	0.0331
LINE215	46.3	9.4	97.7	0.0306
LINE216	46.8	7.8	61.7	0.0338
LINE217	40.2	9.2	107.0	0.0297
LINE218	40.5	8.2	76.3	0.0265
LINE219	36.5	9.1	73.7	0.0258
LINE220	41.4	7.4	67.0	0.0327
LINE221	47.1	9.4	86.0	0.0304
LINE222	38.9	9.6	126.0	0.0276
LINE223	38.5	6.7	103.0	0.0285
LINE224	39.2	7.3	67.3	0.0306
LINE225	35.0	7.1	80.3	0.0281
LINE226	37.4	7.8	82.7	0.0276
LINE227	30.5	7.0	84.7	0.0182
LINE228	33.5	7.2	27.7	0.0280
LINE229	43.4	7.1	81.7	0.0302
LINE230	35.5	7.7	74.7	0.0294
LINE231	NN	0.0	NN	NN
LINE232	42.3	9.1	114.7	0.0268
LINE233	39.7	9.2	102.0	0.0287
LINE234	NN	NN	NN	NN
LINE235	33.2	7.8	95.7	0.0335
LINE236	33.6	7.1	79.3	0.0251
LINE237	41.8	7.3	68.3	0.0303
LINE238	42.4	8.1	95.0	0.0284
LINE239	47.0	8.5	173.7	0.0278
LINE240	38.1	8.1	79.3	0.0322
LINE241	33.2	6.0	80.0	0.0304
LINE242	27.6	6.0	113.3	0.0344
LINE243	33.0	6.1	105.3	0.0262
LINE244	36.8	8.3	157.0	0.0285
LINE245	40.2	7.3	102.0	0.0418
LINE246	32.2	7.4	213.7	0.0227
LINE247	33.1	6.1	112.7	0.0311
LINE248	29.7	8.2	85.3	0.0264
LINE249	26.2	5.6	85.7	0.0235
LINE250	37.7	7.8	57.0	0.0247
LINE251	41.7	6.8	77.0	0.0312
LINE252	28.7	6.9	159.0	0.0230
LINE253	35.1	7.5	79.7	0.0315
LINE254	38.5	7.1	150.0	0.0302
LINE255	28.6	7.2	126.7	0.0294
LINE256	38.9	7.5	108.7	0.0273
LINE257	37.0	7.0	155.0	0.0250
LINE258	41.8	9.0	88.3	0.0348
LINE259	44.1	8.0	118.7	0.0262
LINE260	44.1	9.5	87.3	0.0281
LINE261	34.6	7.0	165.7	0.0234
LINE262	39.7	6.4	78.7	0.0380

**Table A2:** The qRT-PCR primers for the candidate genes.

Candidate Gene (IWGSC1.0)	Candidate Gene (IWGSC2.1)	Forward Primers (5′-3′)	Reverse Primers (5′-3′)
*TraesCS1B01G356600*	*TraesCS1B03G0973300*	AGGCTTTCTTGAAGCCCAGA	GACCAGCATCCTGTCTCCTT
*TraesCS1B01G373900*	*TraesCS1B03G1018500*	GCCCTCAAGGTGAAAGGTCT	CTTCCAGAGCTGCAAGTCG
*TraesCS3A01G406200*	*TraesCS3A03G0950800*	CATGCGGTGCAACTACTACC	GTGTCGCCGATGTTGATGAC
*TraesCS3A01G416300*	*TraesCS3A03G0969200*	CATGAGCCTCAACGAGAAGC	CTCTCATGCTGGTACGACGA
*TraesCS4A01G326400*	*TraesCS4A03G0812600*	AGCAGAACCACCACCTACC	CTGGTGTCGAACGGAAGAAC
*TraesCS4D01G001900*	*TraesCS4D03G0002800*	AGGAATTTGAGGCTGAAGCG	GGGACGTTGGAGTCGTAGTA
*TraesCS4D01G002400*	*TraesCS4D03G0003700*	GACACTGGAGTGGAACTGGA	CTAACAGCGGTCATCAAGGC
*TraesCS5D01G285900*	*TraesCS5D03G0654200*	CTGAGTCTGGTAGCGTGGAG	CCGTCGTTGCCCACGCCGTC

**Table A3:** The DRSA-related traits in the 108 wheat varieties and the genotype data for the developed KASP markers.

Name	*Kasp_1BL_DDRW*	DDRW (g)	*Kasp_5D_DDRW*	*Kasp_3AL_DTRL*	DDRT (mm)	*Kasp_4DS_DNRT*	DNRT	*Kasp_4AL_DTRS*	DTRS (cm^2^)
Aikang 58	GG	0.0304	CC	AA	74.0	CC	100.2	GG	7.7
Bainong 3217	AA	0.0316	TT	AA	54.1	CC	18.6	AA	3.1
Bainong 64	AA	0.0358	TT	AA	88.3	TT	115.2	GG	9.2
Bima 4	GG	0.0198	TT	GG	48.9	TT	11.7	GG	2.4
Gaoyou 503	AA	0.0291	TT	AA	58.6	CC	57.6	GG	5.2
Gaocheng 8901	AA	0.0316	TT	AA	90.5	CC	123.5	GG	10.4
Huapei 5	GG	0.0235	TT	AA	61.5	CC	22.7	GG	3.6
Huaimai 18	AA	0.0251	TT	GG	64.5	TC	69.5	GG	5.9
Huaimai 20	GG	0.0287	TT	AA	80.2	CC	48.8	AA	6.1
Huaimai 21	GG	0.0345	CC	GG	64.0	TT	16.7	AA	3.2
Jimai 19	GG	0.0313	CC	GG	86.1	CC	170.8	AA	11.4
Jimai 20	AA	0.0324	TT	GG	62.9	CC	69.8	GG	6
Jimai 21	GG	0.0222	TT	AA	46.8	CC	10.8	AA	2.2
Jimai 22	GG	0.0307	CC	GG	59.6	CC	103.9	AA	6.3
Jinan 13	AA	0.0299	TT	AA	77.9	TT	23	AA	4.1
Jinan 17	GG	0.0233	TT	AA	36.8	CC	14.4	AA	2.1
Jining 16	AA	0.0196	TT	AA	44.8	CC	10.3	AA	2.1
Jishi 02-1	AA	0.0259	TT	GG	36.0	CC	8	GG	1.7
Jinmai 61	AA	0.0384	TT	AA	66.5	CC	44.9	GG	4.8
Lankao 24	GG	0.0279	TT	GG	61.6	TT	30.9	AA	4.2
Lankao 2	GG	0.0252	TT	AA	69.5	TT	162.5	AA	9.4
Lankao 906	AA	0.0326	TT	AA	54.9	TT	58.3	AA	5.3
Liangxing 66	AA	0.0361	CC	AA	92.7	CC	234	AA	12.8
Liangxing 99	GG	0.0364	CC	GG	61.1	CC	18.6	GG	3.3
Linhan 2	AA	0.0247	TT	AA	79.5	TT	49.6	GG	5.8
Linkang 12	AA	0.0265	TT	AA	63.9	TT	62	AA	5.6
Linmai 2	GG	0.0184	TT	GG	58.6	CC	55.8	GG	5
Linmai 4	AA	0.0245	TT	AA	45.1	CC	47.8	AA	4
Lumai 15	AA	0.0271	TT	GG	57.9	CC	23.7	GG	3.6
Lumai 21	GG	0.0203	TT	AA	54.3	CC	79.4	GG	5.8
Lumai 23	AA	0.0283	TT	AA	76.1	CC	70.1	AA	6.8
Lumai 5	GG	0.0118	CC	GG	60.7	TT	23.9	AG	3.6
Lumai 6	AA	0.024	TT	AA	63.1	CC	61.7	AA	5.6
Lumai 7	AA	0.0297	TT	AA	70.3	CC	68	GG	6.6
Lumai 8	AA	0.0357	TT	GG	62.0	CC	24.8	GG	3.9
Lumai 9	GG	0.0184	TT	AA	81.6	CC	150.8	GG	10.9
Luyuan 502	AA	0.0243	TT	AA	51.8	CC	129.4	GG	7.1
Luohan 2	AA	0.023	TT	GG	61.9	TT	79	AA	6.8
Luomai 21	GG	0.0271	TT	AA	66.7	CC	122.5	GG	8.9
Neixiang 188	AA	0.028	TT	GG	58.1	TT	85	GG	5.9
Neixiang 5	AA	0.019	TT	AA	63.2	CC	77.8	AA	6.8
Shannong 20	AA	0.0232	TT	GG	63.7	TT	166.8	GG	9.5
Shan 150	AA	0.028	TT	AA	67.1	TT	72.2	GG	6.8
Shan 229	AA	0.0294	TT	AA	89.5	CC	140	GG	10.5
Shan 253	AA	0.0202	TT	AA	69.5	CC	295.4	GG	12.2
Shan 354	AA	0.022	TT	GG	55.0	CC	66.9	AA	5.9
Shan 512	AA	0.0226	TT	AA	47.3	CC	71	AA	5
Shan 715	GG	0.0316	CC	GG	72.0	CC	80	AA	7
Shanmai 94	AA	0.0158	TT	AA	59.7	TT	224.4	AA	11.3
Shannong 78-59	AA	0.0279	TT	GG	45.2	TT	64.4	AA	5
Shanyou 225	AA	0.0219	TT	GG	68.0	TT	90.7	AA	7.1
DK171	GG	0.0214	TT	AA	62.3	CC	54.5	GG	5.4
Shijiazhuang 15	AA	0.0252	TT	AA	65.4	CC	31.4	AA	4.3
Shixin 733	GG	0.0199	TT	GG	63.0	TT	60.5	AA	5.9
Shiyou 17	AA	0.0246	TT	AA	68.2	CC	83.2	GG	7.1
Sunong 6	AA	0.0237	TT	AA	69.8	CC	199.5	GG	11.6
Taishan 5	GG	0.0288	TT	AA	64.5	TT	177.3	GG	8.8
Wanmai 19	AA	0.0183	TT	AA	62.5	CC	228.1	GG	11.3
Wanmai 29	AA	0.026	TT	AA	47.6	CC	235.6	GG	8.4
Wanmai 38	AA	0.0208	TT	AA	77.1	CC	190.9	GG	11.8
Wanmai 50	AA	0.017	TT	AA	70.0	CC	293.3	GG	13.8
Wanmai 52	GG	0.0212	TT	AA	58.6	CC	99	GG	7.1
Wanmai 53	AA	0.0213	TT	AA	71.6	CC	140.1	GG	9.5
Wennong 14	AA	0.0211	TT	AA	47.8	CC	156.2	AA	7.7
Wennong 5	AA	0.025	TT	AA	71.3	CC	159.4	GG	10.2
Wunong 148	AA	0.023	TT	AA	50.1	TT	23.7	AA	3.2
Xinnong 291	AA	0.0372	TT	AA	71.3	TT	39	GG	5
Xiaoyan 22	AA	0.0224	TT	GG	63.6	CC	128.8	AA	8.1
Xiaoyan 54	GG	0.023	TT	AA	60.2	CC	102.1	AA	5.6
Xiaoyan 6	AA	0.0287	TT	AA	46.3	CC	119.9	AA	8.6
Xiaoyan 81	AA	0.026	TT	AA	75.6	CC	91.2	AA	6.9
Xinmai 19	AA	0.0231	TT	AA	68.7	CC	108	GG	9
Xinmai 9	GG	0.0268	CC	GG	81.0	TT	12.7	AG	2.8
Yannong 18	AA	0.0223	TT	AA	61.3	TT	68.1	GG	6
Yannong 19	GG	0.0244	TT	AA	64.9	CC	99.2	GG	6
Yanzhan 4110	GG	0.0356	CC	GG	52.6	TC	54.6	GG	5.8
Yumai 18	AA	0.0268	TT	AA	67.6	CC	155.8	AA	8.6
Yumai 21	AA	0.0293	TT	AA	58.9	CC	42.7	AA	5.3
Yumai 2	AA	0.0224	TT	AA	75.3	CC	62.4	AA	5.5
Yumai 34	AA	0.0254	TT	AA	60.6	TT	168.9	GG	9.2
Yumai 35	AA	0.0363	TT	AA	61.2	TT	44.9	GG	5.4
Yumai 47	GG	0.0363	CC	GG	68.8	CC	76.1	AG	5.6
Yumai 49	AA	0.0194	TT	AA	48.2	TT	169.7	GG	7.1
Yumai 50	AA	0.0151	TT	AA	49.3	CC	9.7	AA	1.9
Yumai 63	AA	0.02	TT	GG	37.9	CC	97.3	AA	7.2
Yumai 7	AA	0.0218	TT	AA	59.1	CC	80.5	GG	6.2
Zheng 9023	AA	0.0227	TT	AA	53.5	CC	58.9	AA	4.5
Zhengmai 366	GG	0.0318	TT	GG	41.8	CC	74.1	GG	5.6
Zhengyin 1	AA	0.0237	TT	AA	56.8	TT	181.6	GG	8.6
Zhengzhou 3	AA	0.0302	TT	AA	63.4	TC	140.2	AA	9.4
Zhongmai 871	AA	0.0235	TT	GG	77.7	CC	306.2	GG	10.8
Zhongmai 875	AA	0.0266	TT	AA	52.3	CC	93.6	GG	7
Zhongmai 895	GG	0.026	TT	GG	61.3	CC	148.6	GG	9.2
Zhongyu 5	AA	0.0259	TT	AA	65.3	TT	94.4	GG	7.1
Zhou 8425B	AA	0.0262	TT	AA	58.1	TT	68.7	GG	6
Zhoumai16	GG	0.0182	TT	AA	57.9	CC	58	GG	5.5
Zhoumai 18	AA	0.0236	TT	AA	59.1	CC	13.3	GG	2.6
Zhoumai 19	AA	0.0252	TT	GG	52.4	CC	40.5	GG	3.9
Zhoumai 22	AA	0.0197	TT	GG	47.5	TT	60.9	GG	4.7
Zhoumai 23	GG	0.0181	TT	GG	46.7	CC	88.5	GG	5.3
Zhoumai 25	AA	0.0155	TT	GG	47.8	TT	74.1	GG	4.5
Zhoumai 26	GG	0.0166	CC	GG	40.6	CC	133.9	GG	8.4
Zhoumai 28	AA	0.0195	TT	AA	56.7	CC	94.6	GG	5.6
Zhoumai 30	GG	0.015	TT	GG	46.2	TT	107.8	AA	5.5
Zhoumai 31	AA	0.0162	TT	GG	44.4	CC	36.8	GG	3.8
Zhoumai 32	GG	0.0178	TT	AA	48.1	CC	127	AA	9
Zimai 12	AA	0.0263	TT	AA	73.7	TT	50	AA	5.6
Zixuan 2	AA	0.0224	TT	AA	67.8	CC	141.5	GG	7.9
